# The Diagnostic Ability of rs-DWI to Detect Subtle Acute Infarction Lesion in the Different Regions of the Brain and the Comparison between Different b-Values

**DOI:** 10.1155/2018/7069192

**Published:** 2018-06-05

**Authors:** Tanoj Bahadur Singh, Liwu Zhang, Xiaoting Huo, Guoping Liu, Hongyan Ni, Shun Zhang, Wenzhen Zhu, Jianzhong Yin

**Affiliations:** ^1^First Center Clinical College, Tianjin Medical University, Tianjin 300192, China; ^2^Radiology Department, Tianjin First Central Hospital, Tianjin 300192, China; ^3^Neurology Department, Tianjin First Central Hospital, Tianjin 300192, China; ^4^Radiology Department, Tongji Hospital, Wuhan 430030, China

## Abstract

**Objective:**

To evaluate the diagnostic ability of rs-DWI to detect subtle acute infarction lesion in the different regions of the brain in comparison to routine DWI and the comparison between different b-values.

**Method:**

35 acute brain infarction patients were included. The subtle acute infarction lesions in ss-DWI and rs-DWI sequence were evaluated in 9 anatomical regions of the brain, and the ss-EPI DWI was also acquired with different b-values of 0, 1000, 2000, and 3000s/mm^2^. The McNemar test was performed for comparing the diagnostic ability of ss-DWI and rs-DWI and different b-values. Sensitivity, specificity, positive predictive value (PPV), and negative predictive value (NPV) for the whole brain and in each anatomical region were calculated.

**Result:**

A total of 406 subtle acute infarction lesions were confirmed. The ss-DWI detected 338 subtle lesions, out of which 318 were true positive and 20 were false positive lesions. The rs-DWI detected 386 subtle lesions, out of which 385 were true positive lesions and 1 was true negative lesion. Sensitivity, specificity, positive predictive value, and negative predictive value in rs-DWI were better than ss-DWI in all anatomical regions of the brain. In the comparison of different b-values, b2000 was found better among b1000, b2000, and b3000.

**Conclusion:**

The rs-DWI offers a useful alternative to routine DWI for detecting the subtle acute infarctions, especially in the regions that are susceptible to distortion as in frontal cortex. In addition, high b-value can also provide benefit by increasing diffusion weighting but further raising can deteriorate image quality as SNR is decreased.

## 1. Introduction

Diffusion Weighted Imaging (DWI) is widely used in clinical settings for assessing various brain lesions and particularly plays an important role in the detection of brain infarction [1–3]. In clinical practice, single-shot EPI (ss-EPI) is the sequence of choice to acquire DWI. However, ss-EPI has certain limitations as it produces susceptibility artifacts which manifest as blurring and geometric distortion, particularly at tissue-air interface like skull base [4–6]. Susceptibility artifacts are more prominent at 3T because of its long echo-planar imaging in readout direction and corresponding low bandwidth per pixel in the phase encoding direction which can result in a false negative or false positive result [7]. Therefore, there is a higher possibility for misdiagnosis of subtle lesions. The parallel imaging can help to reduce these artifacts. However, the distortion can only be reduced to a certain point at low resolution [2]. Another solution can be multishot echo-planar imaging, which can also reduce blurring and geometric distortion over ss-EPI. However, even a slight physiological motion can cause profound ghosting artifact [4]. But high-resolution DWI using readout-segmented echo-planar imaging (rs-EPI) can address these issues [5].

The rs-EPI is a newer technique to obtain DW-MRI. In ss-EPI, the whole k-space acquired for a given slice follows a single RF excitation [8, 9], but rs-EPI has multishot RF pulses that sample a subset of k-space points in the readout direction at each shot [1]. The rs-EPI also increases the k-space velocity for each RF shot compared with ss-EPI by shortening the trajectory along the readout direction, hence, decreasing distortion [4]. Further, improvement in rs-EPI can be done by implementing parallel imaging method like GRAPPA (Generalized Autocalibrating Partial Parallel Acquisition) along with 2D navigator [2, 3, 10]. And the signal-to-noise ratio (SNR) is also improved in rs-EPI [7]. Therefore, the detection of subtle infarction lesions, especially at air-tissue interface areas can be improved.

The DWI parameters such as b-value might also improve detection of acute subtle infarcts [11]. With the recent advent of more powerful gradient hardware, it is possible to generate greater b-value and thereby obtain higher diffusion sensitivity [12]. Previous studies have also suggested that high b-value DWI shows superiority to standard b-value DWI performed at 1000s/mm2 in revealing acute cerebral ischemic lesion [13, 14].

Therefore, the purpose of this study was to evaluate the diagnostic ability of rs-DWI to detect subtle acute infarction lesions in the different regions of the brain in comparison to routine DWI and the comparison between different b-values.

## 2. Materials and Methods

### 2.1. Study Population

We conducted the prospective cohort study and included the patients who met the following inclusion criteria: (a) having typical clinical syndromes (dizziness, severe headache, mouth deflection, sudden numbness or weakness, difficulty of speaking or understanding speech, visual disorders, or impaired consciousness) with definite acute infarcts on MRI; (b) interval between the onset of the symptoms and MRI examination < 72 hours; (c) ss-DWI with higher b-value and rs-DWI obtained with adequate imaging quality.

We excluded the patients under following criteria: (a) MR imaging contraindications or claustrophobia; (b) chronic or insidious or unknown onset of stroke attack; (c) motion artifacts on MR images; (d) patients with cerebral hemorrhage, brain tumors, degenerative brain diseases, craniocerebral trauma, postcraniocerebral operation, dyspnea, coma, or other systemic diseases.

This study was approved by the local institutional ethics committee and the informed consent was obtained from each patient or their relative.

### 2.2. MRI Protocols

All patients underwent MRI on Siemens 3T TIM Trio system utilizing an 8-channel head coil.

The performed MRI sequence includes the following: (a) axial T2-weighted image (T2WI) with following parameters: TE:93 ms; TR: 5000 ms; slice thickness: 5 mm; FOV: 220×220 mm^2^; flip angle: 120°; (b) axial T1-weighted image (T1WI) with following parameters: TE: 9.2 ms; TR: 2510 ms; slice thickness: 5 mm; FOV: 220×198 mm^2^; flip angle: 130°; (c) ss-EPI DWI with b-values of 0, 1000, 2000, and 3000s/mm2 and calculated maps of the apparent diffusion coefficient (ADC) with the following parameters: TE: 96 ms; TR: 4000 ms; slice thickness: 5 mm; FOV: 240×228 mm^2^; matrix size: 192×192; acceleration factor: 2; averages: 3; readout bandwidth: 1342Hz; diffusion directions: 3; scan time: 1:36 minutes; (d) rs-EPI DWI with b-values of 0 and 1000s/mm2 and calculated ADC maps with the following parameters: TE: 77 ms; TR: 3100 ms; slice thickness: 5 mm; FOV: 221×210mm^2^; matrix size: 384×384; acceleration factor: 2; averages: 1; readout bandwidth: 310Hz; diffusion directions: 3; number of shots: 15; scan time: 3:54 minutes. A parallel imaging technique with GRAPPA and 2D navigator based reacquisition was used.

### 2.3. Image Analysis

A second-year radiology resident evaluated the lesions in ss-DWI and rs-DWI sequence. During analysis, patients history, other image sequences, and diagnosis were blinded. For the assessment of lesions, 9 anatomical regions of the brain were assessed, which are (1) frontal cortex (FC), (2) parietal cortex (PC), (3) insular cortex (IC), (4) temporal cortex (TC), (5) occipital cortex (OC), (6) white matter (WM), (7) basal ganglia and thalamus (BG), (8) brain stem (BS), and (9) cerebellum (C). Only subtle lesions which measured ≤ 0.5cm and were with adequate conspicuity were included in this study. The total number of such subtle lesions in each region of brain on ss-DWI with b-values 1000, 2000, and 3000 and rs-DWI was counted and recorded.

For the reference standard, a certified neuroradiologist with 15 years of experience went through all the sequences which include T1WI, T2WI, ss-DWI, and rs-DWI to make a diagnosis of each lesion as a true infarction or not.

### 2.4. Statistical Analysis

Statistical analysis was performed by using SPSS software (SPSS, Version 16, SPSS Inc., Chicago, IL, USA). McNemar's test was performed for comparing the overall result of ss-DWI and that of rs-DWI; P<0.05 was set to be statistically significant. Sensitivity, specificity, positive predictive value (PPV), and negative predictive value (NPV) at each anatomical region and the whole brain for ss-DWI, rs-DWI, and also b-1000s/mm^2^, b-2000s/mm^2^, and b-3000s/mm^2^ were calculated.

## 3. Result

### 3.1. Patients

35 patients meeting the inclusion criteria were included, where 26 were male and 9 were female and the mean age was 60.2 years ± 11.01 years (range, 33 years–81 years). The average time from syndrome onset to MR examination was 41.4 hours ± 20.76 hours (4-72hrs) (Table 1).

### 3.2. Subtle Lesions between rs-DWI and Routine ss-DWI

**Figure 1:**
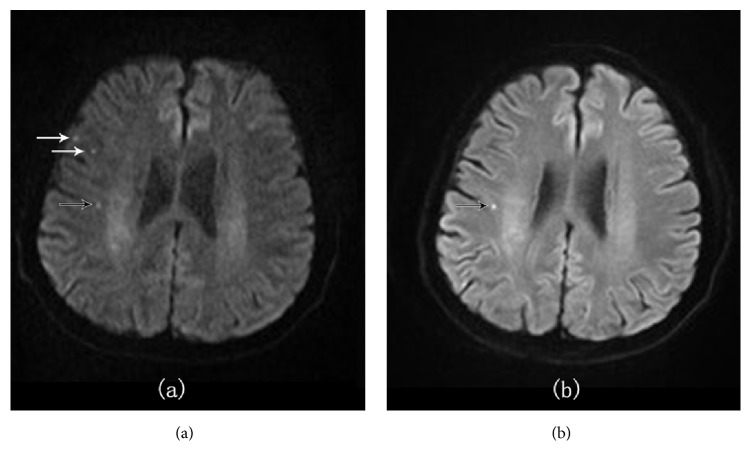
A 49-year-old male patient with acute cerebral infarction. (a) There are 3 suspicious subtle lesions at the right frontal cortex on routine ss-DWI. (b) Only 1 subtle lesion (black arrow) appeared in rs-DWI. Two lesions (white arrows) on ss-DWI did not appear in rs-DWI. rs-DWI showed increased specificity to ss-DWI.

**Figure 2:**
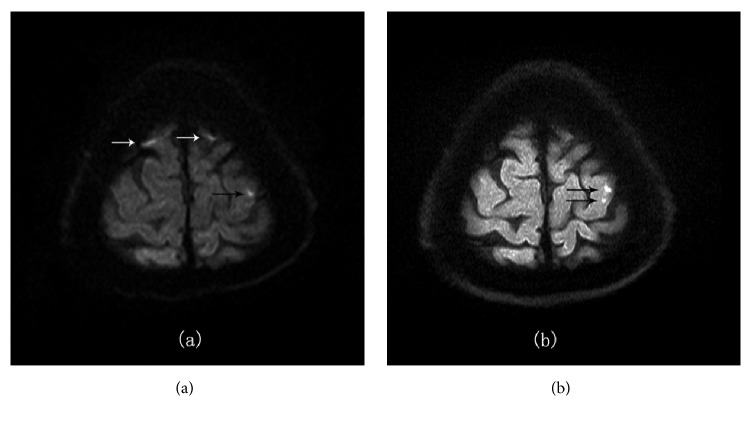
A 46-year-old male patient with acute cerebral infarction showed increased specificity and sensitivity of rs-DWI. (a) On ss-DWI image, there were 3 suspicious prominent subtle lesions. (b) On rs-DWI image, 2 lesions with white arrow did not appear, which should be artifacts from skull, whereas one lesion was confirmed to be true; even another small lesion was also found (black arrows).

**Figure 3:**
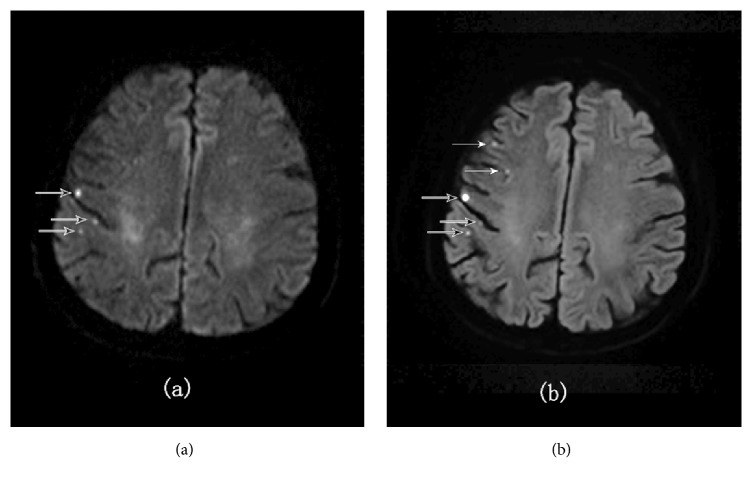
A 49-year-old male patient with acute cerebral infarction showed increased sensitivity of rs-DWI. (a) On ss-DWI image, 3 subtle lesions (black arrows) were found: 1 lesion was at the right frontal cortex, and 2 lesions were at the right parietal cortex. (b) On rs-DWI image, 2 more subtle lesions (white arrows) were found.

In our study, A total of 406 subtle acute infarction lesions were confirmed by the reference standard. The ss-DWI detected a total of 338 subtle lesions, out of which 318 were true positive lesions and 20 were false positive lesions. However the rs-DWI detected 386 subtle lesions, out of which 385 were true positive lesions and 1 was true negative lesion (Table 2). 19 suspicious lesions in ss-DWI were not seen in rs-DWI (Figures 1 and 2) and 67 lesions which were obvious in rs-DWI were very hard to delineate in ss-DWI (Figures 2 and 3). Sensitivity, specificity, positive predictive value (PPV), and negative predictive value (NPV) of rs-DWI were found better than ss-DWI (Table 3). Sensitivity, specificity, PPV, and NPV of rs-DWI were, respectively, 99.7%, 95%, 99.7%, and 95% and ss-DWI was, respectively, 82.38%, 0%, 94%, and 0%. We had found very high sensitivity and PPV and relatively higher specificity and NPV in rs-DWI. Moreover, McNemar test showed rs-DWI was related to reference standard (p=1, p>0.05), whereas ss-DWI was significantly different from a reference standard (p<0.05).

### 3.3. Subtle Lesions in Different Regions of Brain

Table 4 showed the statistical analysis at the different anatomical regions of the brain. Moreover, most of the lesions were found in frontal cortex and white matter. Total of 97 lesions in ss-DWI and 110 lesions in rs-DWI were found in frontal cortex and total of 88 lesions in ss-DWI and 112 lesions in rs-DWI were found in white matter. In white matter, rs-DWI has detected 27 more true positive lesions than ss-DWI and no false positive lesions were detected by rs-DWI while 3 false positive lesions were detected by ss-DWI. Similarly, in the frontal cortex rs-DWI has detected 22 more true positive lesions than ss-DWI and as in the white matter no false positive lesion was found in rs-DWI while 9 false positive lesions were found in ss-DWI. Sensitivity, specificity, positive predictive value, and negative predictive value of rs-DWI looked better than ss-DWI in all regions. Sensitivity difference in white matter between rs-DWI and ss-DWI (24.2%) was slightly more than in frontal cortex (20%) while specificity difference in frontal cortex (100%) was obviously higher than in white matter (41.6%).

### 3.4. Subtle Lesions between Different b-Values

**Figure 4:**
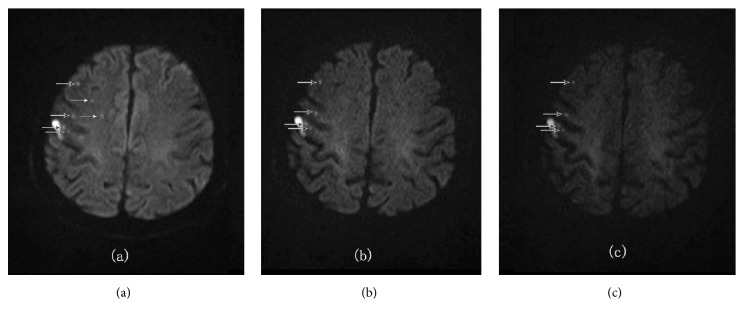
A 49-year-old male patient with acute cerebral infarction showed increased specificity for high b-value. (a) On ss-DWI image with b1000, 6 subtle infarcts were found. With (b) ss-DWI image with b2000 and (c) ss-DWI image with b3000, only 4 lesions (black arrows) were confirmed and 2 lesions with white arrows were false positive at b1000.

The total number of subtle infarction lesions detected at b1000, b2000, and b3000 were 338, 336, and 327, respectively. True positive and false positive lesions detected at b1000 were, respectively, 318 and 20, at b2000 were, respectively, 331 and 5, and at b3000 were, respectively, 319 and 8 (Table 5). 15 more false positive lesions were detected at b1000 compared with b2000 as well as 12 more false positive lesions compared with b3000 (Figure 4). 13 more true positive lesions were detected at b2000 compared with b1000, as well as 12 more true positive lesions compared with b3000. Sensitivity, specificity, positive predictive value, and negative predictive value of b1000 were found, respectively, to be 82.3%, 0%, 95.7%, and 0%, of b2000 were found, respectively, to be 85.7%, 75%, 98.5%, and 21.4%, and of b3000 were found, respectively, to be 82.6%, 60%, 97.5%, and 15.1%. b2000 was found to be better in sensitivity, specificity, PPV, and NPV among the three and b3000 to found to be better than b1000.

## 4. Discussion

The identification of small lesions is important in acute stroke patients, especially in transient ischemic attack subjects. DWI sequence is considered as an important imaging sequence for the detection of infarct lesions. The present trend in a clinical setting is the use of ss-DWI but it has a major drawback as it produces susceptibility artifact in the form of distortions and blurring [5]. However, quality of DWI can be significantly improved using rs-DWI sequences [1]. In this study, 113 true small infarcts which were missed by ss-DWI were detected by rs-DWI, revealing that rs-DWI is a more effective technique for the diagnosis of subtle infarcts. Table 2 shows that in cortical area and moreover in the frontal cortex, rs-DWI has detected more true lesions than ss-DWI. Actually, air-tissue interface areas, like the skull base, orbit, and paranasal sinuses, are more prone to susceptibility artifact [15] and such artifact is more pronounce in the cortical region which is close to the skull. This could result in a misdiagnosis of the lesions. In our study, 19 suspicious lesions were also found in ss-DWI which were not seen in rs-DWI (Figures 1 and 2). In rs-EPI, the k-space is covered with several multishot RF segmentations in the readout direction [5]. It is already shown in previous studies that rs-EPI is superior in SNR and CNR compared to ss-EPI [16]. The magnitude of artifacts in EPI has an inverse relation with the speed with which k-space is traversed along the phase encoding direction [4]. In rs-EPI readout, the length is less which results in an increase in k-space velocity along the phase direction. Hence, artifacts in rs-DWI are decreased compared to ss-DWI [17]. The motion-induced phase error dealt with the approach by correcting linear and nonlinear phase errors through the use of a 2D navigator echo [18]. Therefore, the resolution, SNR, and CNR of DWI with rs-EPI are totally improved. The better resolution and improved SNR and CNR have led to detecting small lesions more accurately and visualizing anatomical details more precisely.

The rs-DWI can provide a wider range of benefit in clinical practice [2, 16]. Artifacts that could be mistaken for pathological brain lesion are reduced and the probability of confusion between enhancing lesions with susceptibility artifacts is diminished. In addition to detection of subtle infarct lesion more accurately, the rs-DWI can also be useful in detection and differentiation of other lesions in brain providing additional pathological details particularly in the skull base, posterior fossa, orbits, and nasal cavity. These areas are often nondiagnostic and overlooked by routine ss-DWI [2, 3].

In the white matter, the total number of lesions detected was the highest, which was 115 (Table 1). Also, rs-DWI has detected 17 more true positive lesions than ss-DWI. Moreover, the differentiating between acute infarct lesions from the demyelinating lesions might have difficulty sometime with conventional MRI, because both these lesions appear hyperintense in Diffusion Weighted Imaging [19, 20]. However, according to some literature, the ADC and PWI can be helpful in differentiating them. There is a trend for higher ADC in demyelinating lesions when compared to acute ischemic lesions [20, 21] and PWI parameter specially MTT can be really helpful to differentiate them [20]. In our study, higher b-value also showed having benefit in differentiating these two situations, and the acute infarct lesions would keep the high signal whereas the demyelinating lesions decreased the signal while the b-value was increasing.

**Figure 5:**
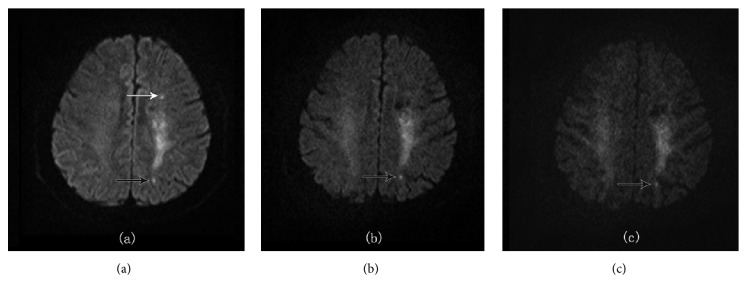
A 47-year-old male patient with acute cerebral infarctions. (a), (b), and (c) were the ss-DWI image with b-values 1000, 2000, and 3000. Two lesions were found at b1000 image. The parietal lesion (black arrow) kept the signal while the b-value was increasing, which should be an infarction lesion. However the frontal lesion (white arrow) decreased the signal while the b-value was increasing, which should be a demyelinating lesion. Noise looked more pronounced in b3000 than b2000.

Table 4 shows DWI obtained at higher b-values (b=2000, b=3000) had higher lesion detection rate with better sensitivity, specificity, PPV, and NPV than b=1000. However, b-2000 looked better than b-3000 in the overall statistical analysis. Use of high b-value in infarction is based on the possibility of increasing the contrast between the areas of restricted water diffusion (typical of recent infarction) and normal brain tissue. As b-value increases, diffusion weighting also increases; therefore, higher b-values better enable the detection of the lesions with subtle diffusion restriction [11, 13]. However, the exponential loss of signal intensity exists as the b-value increases which means SNR decreases both in the lesion and in the contra-lateral normal area for a given voxel with an increasing b-value and is a trade-off for the increased diffusion weighting at higher b-values (Figure 5).

This study has some shortcomings. First, we could not get the pathological results for the brain infarction lesions and a reference standard was used based on an experienced neuroradiologist and all the imaging and clinical information. Second, in the insular cortex, basal ganglia, brainstem, and cerebellum the number of true negative lesions was 0; therefore we could not calculate specificity and negative predictive value.

## 5. Conclusion

This work shows that rs-DWI offers a useful alternative to routine DWI for detecting acute infarction subtle lesions. rs-DWI offers benefits especially in the regions of tissue-air interface that is susceptible to distortion as in frontal cortex. rs-DWI produces less distortion and reduces blurring in a reasonable scan time. The diagnostic sensitivity and specificity using rs-DWI are all improved for detection and exclusion of subtle infarction lesion. In addition, high b-value can also provide benefit by increasing diffusion weighting but further raising can deteriorate image quality as SNR is decreased. Our work showed b2000 was better in sensitivity and specificity than b1000 or b3000.

## Figures and Tables

**Table 1:** The demography of patients.

**Demographic Marker**		**Mean ± SD**
Number of patients		35
Age (years)		60.2 ± 11.01
Sex	Male	26/35 (74.3%)
	Female	9/35 (25.7%)
Time of Onset (hours)		41.45 ± 20.76

**Table 2:** The number of subtle infarcts in each anatomical region on ss-DWI and rs-DWI.

**brain regions**	**ss-DWI**		**rs-DWI**		**Reference Standard**	
	True Positive	False Positive	True Positive	False Positive	Positive Lesion	Negative lesion
Frontal Cortex	88	9	110	0	110	9
Parietal Cortex	55	4	61	0	62	4
Insular Cortex	4	0	6	0	6	0
Temporal Cortex	25	2	27	0	27	2
Occipital Cortex	30	2	32	0	32	2
White Matter	85	3	112	1	112	3
Basal Ganglia	16	0	17	0	17	0
Brain Stem	4	0	4	0	4	0
Cerebellum	11	0	16	0	16	0

**Total**	318	20	385	1	386	20

ss-DWI: single shot-Diffusion Weighted Imaging; rs-DWI: readout segment-Diffusion Weighted Imaging.

**Table 3:** Statistical analysis of the diagnostic ability between ss-DWI and rs-DWI in the whole brain.

**Sequence**	**Results**				**P-Value ** **(McNemars)**	**Statistical Analysis (%)**			
	True Positive	True Negative	False Positive	False Negative		Sensitivity	Specificity	PPV	NPV
**rs-DWI**	385	19	1	1	P=1	99.7	95	99.7	95
**ss-DWI**	318	0	20	68	P≤0.001	82.38	0	94	0

ss-DWI: single shot-Diffusion Weighted Imaging; rs-DWI: readout segment-Diffusion Weighted Imaging; PPV: positive predictive value; NPV: negative predictive value.

**Table 4:** Statistical analysis of the diagnostic ability between ss-DWI and rs-DWI in each anatomical region of the brain.

**Regions**	**True Positive**		**True Negative**		**False Positive**		**False Negative**		**Statistical Analysis (%)**							
									**Sensitivity**		**Specificity**		**PPV**		**NPV**	
	*ss-DWI*	*rs-DWI*	*ss-DWI*	*rs-DWI*	*ss-DWI*	*rs-DWI*	*ss-DWI*	*rs-DWI*	*ss-DWI*	*rs-DWI*	*ss-DWI*	*rs-DWI*	*ss-DWI*	*rs-DWI*	*ss-DWI*	*rs-DWI*
Frontal Cortex	88	110	0	9	9	0	22	0	80	100	0	100	80	100	0	100
Parietal Cortex	55	61	0	0	4	1	7	4	93	98.3	0	100	88.7	100	0	80
Insular Cortex	4	6	0	0	2	0	0	0	66.6	100	N/A	N/A	100	100	N/A	N/A
Temporal Cortex	25	27	0	0	2	0	2	2	92.5	100	0	100	92.5	100	0	100
Occipital Cortex	30	32	0	0	2	0	2	2	93.8	100	0	100	93.8	100	0	100
White Matter	85	112	1	1	3	0	27	2	75.8	100	25	66.6	96.5	99	3	100
Basal Ganglia	16	17	0	0	0	0	1	0	94.1	100	N/A	N/A	100	100	0	N/A
Brain Stem	4	4	0	0	0	0	0	0	100	100	N/A	N/A	100	100	0	N/A
Cerebellum	11	16	0	0	0	0	5	0	68.8	100	N/A	N/A	100	100	0	N/A

ss-DWI: single shot Diffusion Weighted Imaging; rs-DWI: readout segment-Diffusion Weighted imaging; PPV: positive predictive value; NPV: negative predictive value.

**Table 5:** Statistical analysis of the diagnostic ability among b1000, b2000, and b3000.

**b values**	**True Positive**	**True Negative**	**False Positive**	**False Negative**	**Statistical Analysis (%)**			
					**Sensitivity**	**Specificity**	**PPV**	**NPV**
b1000	318	0	20	68	82.3	0	95.7	0
b2000	331	15	5	55	85.7	75	98.5	21.4
b3000	319	12	8	67	82.6	60	97.5	15.1

PPV: positive predictive value; NPV: negative predictive value.

## Data Availability

The data used to support the findings of this study are available from the corresponding author upon request.
